# Might Cortical Hyper-Responsiveness in Aging Contribute to Alzheimer’s Disease?

**DOI:** 10.1371/journal.pone.0105962

**Published:** 2014-09-10

**Authors:** Michael S. Jacob, Charles J. Duffy

**Affiliations:** 1 Department of Neurology and the Center for Visual Science, The University of Rochester Medical Center, Rochester, New York, United States of America; 2 Department of Psychiatry, The University of California San Francisco Medical Center, San Francisco, California, United States of America; University of Rome “Foro Italico”, Italy

## Abstract

Our goal is to understand the neural basis of functional impairment in aging and Alzheimer’s disease (AD) to be able to characterize clinically significant decline and assess therapeutic efficacy. We used frequency-tagged ERPs to word and motion stimuli to study the effects of stimulus conditions and selective attention. ERPs to word or motion increase when a task-irrelevant 2^nd^ stimulus is added, but decrease when the task is moved to that 2^nd^ stimulus. Spectral analyses show task effects on response power without 2^nd^ stimulus effects. However, phase coherence shows both 2^nd^ stimulus and task effects. Thus, power and coherence are dissociably modulated by stimulus and task effects. Task-dependent phase coherence successively declines in aging and AD. In contrast, task-dependent spectral power increases in aging, only to decrease in AD. We hypothesize that age-related declines in signal coherence, associated with increased power generation, stresses neurons and contributes to the loss of response power and the development of functional impairment in AD.

## Introduction

Aging is the #1 risk factor for AD, although mechanisms linking those conditions have long remained obscure [1]. The hypothesis that aging induced heightened activation may trigger the transition to early AD [2] has recently found support in neuro-imaging [3], [4] and molecular studies [5], [6], [7].

We previously found evidence of visual cortical hyper-responsiveness in aging [8], consistent with cellular studies in aged animals which found increased neuronal excitability and diminished selectivity [9], [10]. We consider that age-related cortical hyper-responsiveness may reflect a variety of contributing factors: local disinhibition from intra-cortical (e.g., loss of GABAergic neurons) or cortico-cortical (e.g., fronto-posterior de-afferentation) [11], [12], [13], and over-activation as a consequence of, or in compensation for, signal degradation [14], [15].

We have now examined these hypotheses by assaying competitive attentional control of the dorsal and ventral extrastriate cortical visual systems in aging and AD. These parallel systems partition signals for object and motion processing [16], [17]. The relative activity of these pathways is shaped by selective attention’s biasing their competitive interactions [18], [19], [20] to implement behavioral priorities and optimize function [21], [22], [23].

We have explored the attentional control of visual motion and object processing in monkeys and humans. In monkey single neurons, we found competitive attentional control between pattern and object motion [24]. In human studies, we found that this competition uniquely disrupts perception in early AD [25]. Such attentional control of sensory processing has been seen in ERP amplitudes [26], [27], evoked power [28], [29], [30], and phase coherence [31], [32].

We have found that ERPs reflect attentional and perceptual impairments in AD [33], [34] and now focus on how those changes may distinguish aging and AD. We find that aging degrades response coherence with a paradoxical increase in response power. This cortical hyper-responsiveness is absent in AD, leading us to consider whether aging may stress posterior cortical neurons and contribute to neurodegenerative processes in AD.

## Methods

### Subject Groups

Young normal subjects (YNs, n = 18) were undergraduates at the University of Rochester. Older normal subjects (ONs, n = 17) were from elderly wellness programs or were the spouses of ADs. ADs (n = 14) were from clinical programs at the University of Rochester Medical Center, diagnosed by a neurologist or psychiatrist specializing in dementia within two years of these studies. Written Informed consent, including screening for competency to grant consent, was obtained before subject enrollment. All procedures are approved by the University of Rochester RSRB. That approval covers this work and ongoing studies applying similar neurophysiological methods in human subjects.

All subjects had normal range Snellen visual acuity (monocular at least 20/40) and contrast sensitivity (5 spatial frequencies, 0.5 to 18 cycles/°, VisTech Consultants, Inc., Dayton, OH). AD patients met DSM-IVR criteria for probable AD including: significant memory impairment with signs and symptoms of either aphasia, agnosia, apraxia, inattention, disorganization, or executive dysfunction [35]. All patients would also meet DSMV criteria, whereas no non-patents would satisfy those criteria.

Diagnostic classification was supported by: the Mini-Mental State Examination of global function [36], WMS-Revised (WMS-R) [37] verbal paired associates (immediate and delayed recall), animal naming verbal fluency, money road map test of topographic orientation [38], WMS-R figural and facial memory tests of visual recognition, and line orientation test of spatial relations [39]. These tests yielded scores consistent with group membership (Table S1). We note the relatively mild impairment of our AD subjects on the MMSE, suggesting mild or early Alzheimer’s, corresponding with more pronounced executive impairment at this stage in the disease as indexed by Trails B scores [40], [41], [42].

### Neurophysiological Recordings

Scalp recorded EEG was obtained using a 32-channel Neuroscan system with electrodes in the international 10–20 configuration at impedances <5 kΩ. Activity was low pass filtered at 100 Hz and a high pass filtered at 0.1 Hz and sampled at 500 Hz/32-bit resolution creating MATLAB files. Subjects maintained centered visual fixation (+/−10°) on a centered spot screen during recording. Eye position was monitored using infrared oculometry (ASL, Inc.). EEGLAB created independent components for each subject and recording session to remove eye blinks in one or two components.

### Visual Stimuli

**Figure 1:**
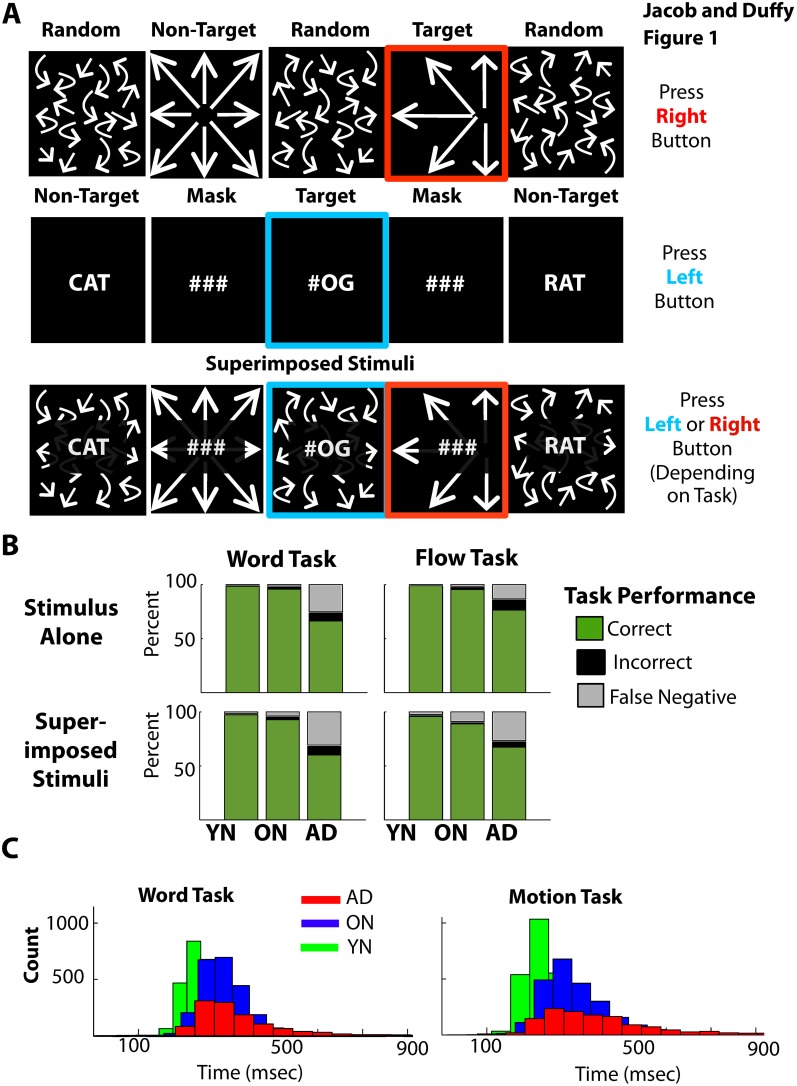
Behavioral paradigm and responses used in these studies. A. Schematic diagram of the visual stimuli and behavioral paradigm. Top: The optic flow stimulus stream consists of radial pattern motion alternating with random dot motion. The radial stimuli present a random series of non-targets with a centered focus of expansion (75%) and targets with a left or right side focus of expansion (25%). Middle: The word object stimulus stream consists of letters alternating with a dot grid. The letter stimuli present a random series of non-target three letter words (75%) and target letter pairs with a left or right side dot grid (25%). Bottom: Superimposed optic flow and word object stimulus streams including a word task left target (blue frame) and a flow task right target (red frame). B. Performance scored by button presses during the word task (left) and flow task (right) with stimuli presented alone at 1.11 Hz (top) or with superimposed stimuli (bottom). Bar graph of percent of total responses to target stimuli scored as correct (green), incorrect (black), and false negative (gray, no response to a target stimulus) for all subject groups (abscissa). ADs showed lower accuracy than the other groups. C. Number of trials (ordinate) yielding the indicated push button response times (abscissa) for all conditions of the word and flow tasks in the three subject groups. ONs and AD showed longer response times then YNs.

Subjects sat facing a rear-projection tangent screen’s 60°×40° image. We presented streams of flow and words to activate dorsal and ventral processing, respectively. Flow alternated with random motion masks, words with pound sign masks, with a 50/50 duty-cycle at 1.11 Hz or 1.57 Hz. Target stimuli (25%) were randomly interspersed with non-targets (75%). (Figure 1A).

Optic flow stimuli contained 2000 white dots on a dark background (Michelson contrast = 0.83 at 60 Hz frame rate). Non-target, optic flow contained a screen centered focus-of-expansion with dot speed increasing with distance from the center. The masking, random motion stimulus contained dots moving at the same average speeds with direction and position randomized to yield 0% pattern coherence. Target optic flow was the same as non-target optic flow, except the focus of expansion was shifted to the left or to the right by 20°.

Word stimuli contained 3 letters occupying the central 4°×12° and alternated with 3 similarly sized modified pound signs (mask). Target word stimuli consisted of 2 letters from a 3-letter word with the first (left) or last (right) letter replaced by the mask. The task required that subjects press the button on the side of the hash mark. For superimposed stimuli, the region between letters was transparent and dot motion was visible. Flow and word stimuli were matched for total number of pixels, luminance, and contrast.

### Behavioral Paradigm

All subjects completed five recording blocks. The first block was two minutes to, “Rest quietly and remain fixated on the screen.” The other blocks engaged subjects in the motion/word task (Figure 1B). Task blocks presented ∼120 target stimuli with subjects told to use the flow or word stimuli to guide left/right button presses which were followed by a beep tones if the correct side, and boops if incorrect. False negatives (no response to targets) and false positives (responses to non-targets) did not yield tones. Error intervals were omitted from the analysis, including false positive responses to non-target stimuli and false negative failures to respond to target stimuli.

### Event-related Potentials Analysis

The continuous dataset for each recording block was divided into 1s epochs (–100 to 900 ms post-stimulus onset), averaged for subjects and groups and low pass filtered at 10 Hz. Epochs were created for all sessions and stimulus conditions including single, superimposed, target and non-targets for flow and word stimuli. All stimuli preceded by correct response were included in the analyses. Grand averages were created for subjects and groups. These were low pass filtered at 10 Hz for display. N2 responses to target and non-target stimuli were identified as the first negative deflection 150–200 ms after stimulus onset.

Group and subject averages were peak detected in MATLAB by finding the time point where the first derivative of the voltage was approximately zero. Individual subject amplitudes were measured as the mean voltage in the 20 ms centered on the group peak latency. P1s were prominent in the ON and AD word responses, making P1N2 amplitudes the most consistent measure across all conditions and groups.

Latencies and amplitudes were derived for P1N2, N2b and P3s for each stimulus, task condition, and subject group and entered in to 3-way ANOVAs. The two stimulus frequencies (1.11, 1.57 Hz) did not effect P1N2, N2b or P3s (F3, 370 = 1.62, p = 0.184) but for shorter P3 latencies at the higher frequency (F3, 370 = 6.10, p<0.001). Analyses of the two different stimulus frequency data sets yield nearly identical results for all reported measures. Thus, our analyses combined these data.

### Spectral Analysis of EEG

Power spectra from Fourier transformation (EEGLAB) were estimated at a frequency resolution of 0.05 Hz. The resting, eyes-open condition was subtracted from the task-recorded spectra. Power at each stimulus fundamental frequency and their harmonics were measured peak to trough at OZ. Three YN subjects, two ON and one AD subject did not complete the two minute eyes-open session and were not included in the spectral analysis. The spectral peak amplitudes were entered into a 3-way analysis of variance to identify main effects of stimulus, task condition, and subject group. Tukey’s Honestly Significantly Different (THSD) post-hoc tests (p<.05) were applied to ascertain the sources of significant effects.

### Time-Frequency Coherence

The time-frequency analyses of inter-trial phase coherence was based-on the continuous data files from each stimulus and task condition [43]. These files were aligned on the onset of the target or non-target stimuli. Phase coherence (EEGLAB) was calculated over 4s windows across log-spaced frequencies from 0.5 to 40 Hz and increasing wavelet cycles from .5 to 1 cycle/Hz. Phase coherence measures the consistency in phase across time/frequency points and trials and is scaled 0 to 1, where 1 represents identical phase coherence across trials. These measures were entered into 3-way ANOVAs to identify effects across stimuli, task condition, and subject group.

## Results

### Behavioral Task Performance

Despite the complexity of the behavioral tasks, all groups perform well with single and superimposed word and flow stimuli (Figure 1) with differences attributable to group membership (Table S1). We assessed task performance in the alone and combined recording blocks, measuring accuracy, as percent correct push-button responses, and response time (RT). The only significant influence on these measures was subject group (MANOVA of accuracy and RT, for word or flow alone vs. with a 2^nd^ stimulus, in the word or flow tasks, at the fast or slow stimulus frequencies, yields a group effect: F4, 328 = 38.5, p<.001; THSDs for accuracy: YNs = ONs > ADs; THSDs for RT: YNs < ONs < ADs). Thus, we considered task difficulty to be fairly well-balanced across the word and flow stimuli and tasks, with single and superimposed stimuli.

### Stimulus, Task, and Group Effects on ERPs

**Figure 2:**
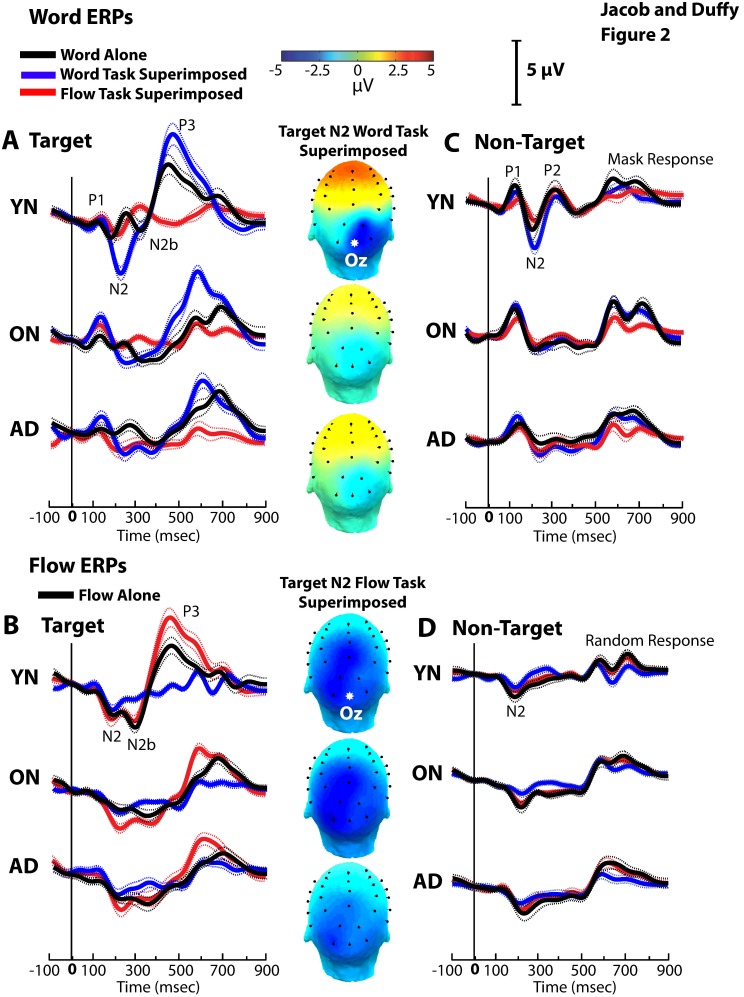
ERP traces and scalp maps of responses. Group average ERPs +/− sem envelope for YNs (top), ONs (middle), and ADs (bottom). A. Responses to target words presented alone (black), include P1, N2, N2b and P3 components. The amplitude of the N2 and P3 components increases with the addition of a task irrelevant flow stimulus superimposed on the word stimulus (blue). The N2 decreases, and the N2b and P3 are eliminated, when the superimposed flow and word stimuli are presented in the context of task change to the flow task (red). Voltage scalp maps (center) with prominent occipital/posterior activation of the N2 response to the word alone stimuli. B. ERPs target optic flow presented alone include N2, N2b and P3 (black) with the P3 increased by adding an irrelevant word stimulus (red). The N2b and P3 are eliminated when switching to the word task (blue). Voltage scalp maps (center) show prominent parietal/posterior activation of the optic flow N2. C. ERPs to non-target words presented alone (black) and the effect of adding an irrelevant motion stimulus (blue) or switching tasks (red). D. ERPs to non-target flow presented alone (black) and the effect of adding an irrelevant word stimulus (red) or switching tasks (blue). The non-target responses after 700 ms illustrate subtler effects of the transition to the masking noise stimulus.

Group averaged ERPs to word and flow stimuli have tri-phasic waveforms at occipital electrodes (Fig. 2, center column). Word P1N2s are largest at Oz, without significant lateralization (O1 vs. O2, Group-by-Electrode Interaction F1, 466 = 1.02, p = 0.313). Flow P1N2s were largest at Oz across groups, but equally so at Pz in YNs (Group-by-Electrode Interaction F1, 666 = 4.90 p<0.001). Peaks at Oz across stimuli and groups led us to focus on that site, but other active sites yield similar results.

We compared P1N2s evoked by target word and flow stimuli, for the three subject groups, recorded in three conditions: 1) word or flow presented alone, 2) word or flow presented with the other stimulus superimposed as a task-irrelevant 2^nd^ stimulus, and 3) word or flow presented with the other stimulus superimposed as the task relevant stimulus.

P1N2 amplitudes are larger with task-irrelevant 2^nd^ stimuli (condition F2, 421 = 6.87, p = .001), especially with flow added to word stimuli (condition-by-stimulus F2, 421 = 12.41, p<.001). Group effects are prominent in N2 latencies, with delays in ONs and ADs (group F2, 421 = 8.1, p<.001), especially to word stimuli (group-by-stimulus F2, 421 = 4.76, p = .009; condition F2, 421 = 14.49, p<.001, THSDs alone < combined; condition-by-stimulus F2, 421 = 7.11, p<.001, THSDs word with flow in word task > others). (Figures 2A and B).

Later response components (the negative and positive components following the N200 response, the N2b and P3) also showed significant effects of stimulus, condition, task, and subject group: N2b amplitudes are largest with flow in YNs (group-by-stimulus F2, 421 = 5.53, p = .004) and delayed in ONs and ADs, especially with words (stimulus-by-group F2, 421 = 3.99, p = .02, THSDs YN flow < others). P3s are also largest in YNs (group F2, 421 = 16.78, p<.001) and delayed in ONs and ADs, especially to flow (group-by-stimulus: F2, 421 = 17.67, p<.001, THSDs all others > YN).

P1N2 amplitudes evoked by non-target stimuli show larger responses to words, especially in YNs (stimulus F1, 416 = 39.52, p<.001, THSDs word > flow; group F2, 416 = 4.35, p = .014, THSDs YN > AD; stimulus-by-group F2, 416 = 6.95, p = .001, THSDs word > flow, YN = ON > AD; stimulus-by-condition F2, 416 = 12.2, p<.001, THSDs word combined > others). Non-target N2 latencies show group and condition effects, with the fastest peak in YNs (condition F2, 416 = 6.01, p = .003, THSDs combined with task > others; group F2, 416 = 4.02, p = .02, THSDs YN < AD). (Figure 2C and D).

Parallel analyses at Pz show a similar pattern of condition and group effects for word responses. At Pz, the flow responses are less distinct, but show the same pattern of relative response amplitudes without statistical significance. Latency effects are the same across Oz and Pz.

Thus, stimulus and task conditions affect word and flow ERPs with unexpectedly larger responses with superimposed task-irrelevant 2^nd^ stimuli, and delayed peaks in the older subject groups. The insensitivity of ERP amplitudes to group is consistent with our previous finding that rapidly repeating flow stimuli minimize group differences [34], [44]. This prompted our use of spectral analyses that are suited to repetitive stimuli.

### Spectral Power Analyses

The use of frequency tagged stimuli enables Fourier analysis of EEG data across the time period of each condition. These analyses reveal task effects, but not 2^nd^ stimulus effects, on the power spectra at the stimulus frequency.

Power spectra for word stimuli, presented alone in the word task, show a peak at the stimulus frequency and at five harmonics with main effects of group but not of a 2^nd^ stimulus (added flow) (MANOVA: group F12, 118 = 2.31, p = .011, THSD: ON > AD, condition p = 0.380, interaction p = 0.895). Power spectra for flow stimuli, presented alone, also show a clear peak at the stimulus frequency, with group effects but not 2^nd^ stimulus effects (MANOVA group F12, 122 = 2.04, p = .026, THSD: ON > YN = AD; condition p = 0.577; interaction p = 0.992).

**Figure 3:**
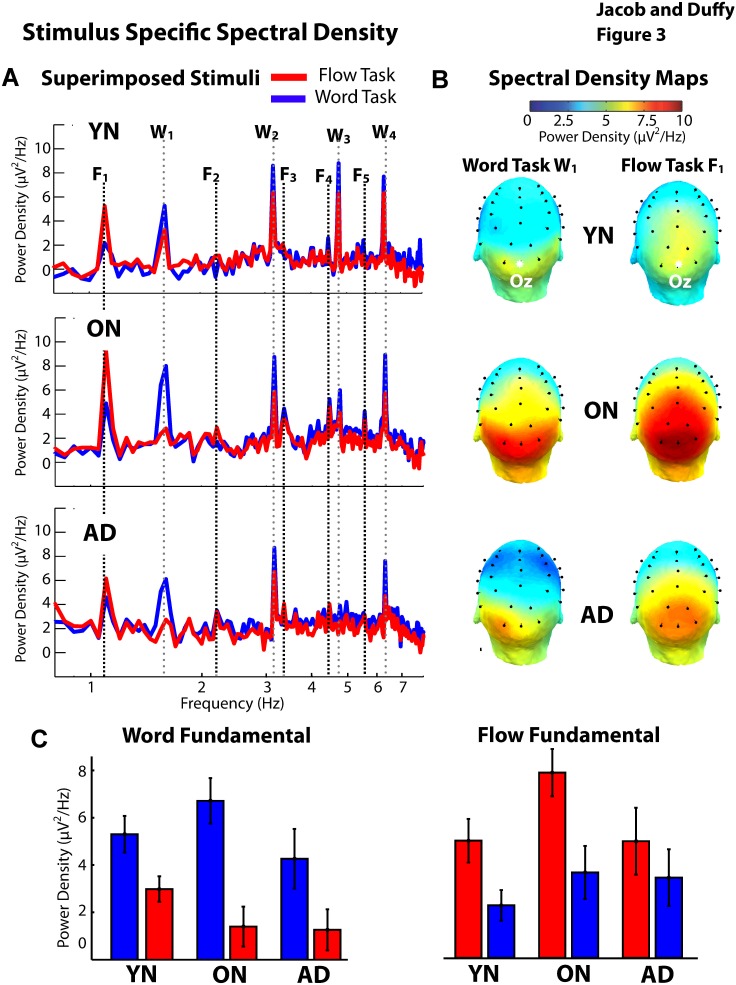
Frequency spectra of scalp recorded electrical activity. Stimulus specific frequency spectra of cortical responses to superimposed optic flow at 1.11 Hz and words at 1.57 Hz during the flow (red) or word (blue) button press tasks. A. Task effects are seen for all groups, with larger responses at the frequency of the task-relevant stimulus. B. Spectral density scalp maps with prominent occipital/posterior activation of the word (left) and flow (right) responses, substantially more evident in ONs than YNs or ADs. C. Bar graphs of spectral power (ordinate, mean +/− sem) show task effects for the word (left) and flow (right) stimuli at the fundamental frequencies, during the flow (red) and word (blue) push button tasks, for the three subject groups (abscissa).

Mirroring our approach to the ERPs, we compared the amplitudes of spectral peaks at the stimulus fundamental frequencies for word and flow stimuli, across the three subject groups, and three stimulus conditions. A three-way ANOVA shows larger peaks to the task-linked stimulus (condition F2, 421 = 13.000, p<0.001; condition-by-stimulus F2, 421 = 33.01, p<0.001, THSDs task-relevant > irrelevant) with the largest peaks in ONs (group F2, 421 = 6.5, p = 0.002; THSDs ON > YN = AD). Task effects were also present in the higher harmonics of the word spectra (1st harmonic p<0.001; 2^nd^ harmonic p = 0.005; 3rd harmonic p = 0.012) but not of the flow spectra. (Figure 3) Again, responses at Pz show the same main effects of task and group, as described above for the spectra recorded at Oz.

Thus, unlike the ERPs, the power spectra show similar task effects on both word and flow responses, but do not show effects of adding a 2^nd^ task-irrelevant stimulus. The spectra also show robust group effects, with the surprising finding of spectral power increasing from YNs to ONs, but decreasing from ONs to ADs.

### Phase Coherence Analyses

We considered that differences between the ERPs and the power spectra might reflect changes in the domain of response phase coherence. We focused our analyses on the frequency range of the stimuli with comparisons across stimuli, task conditions, and subject group.

**Figure 4:**
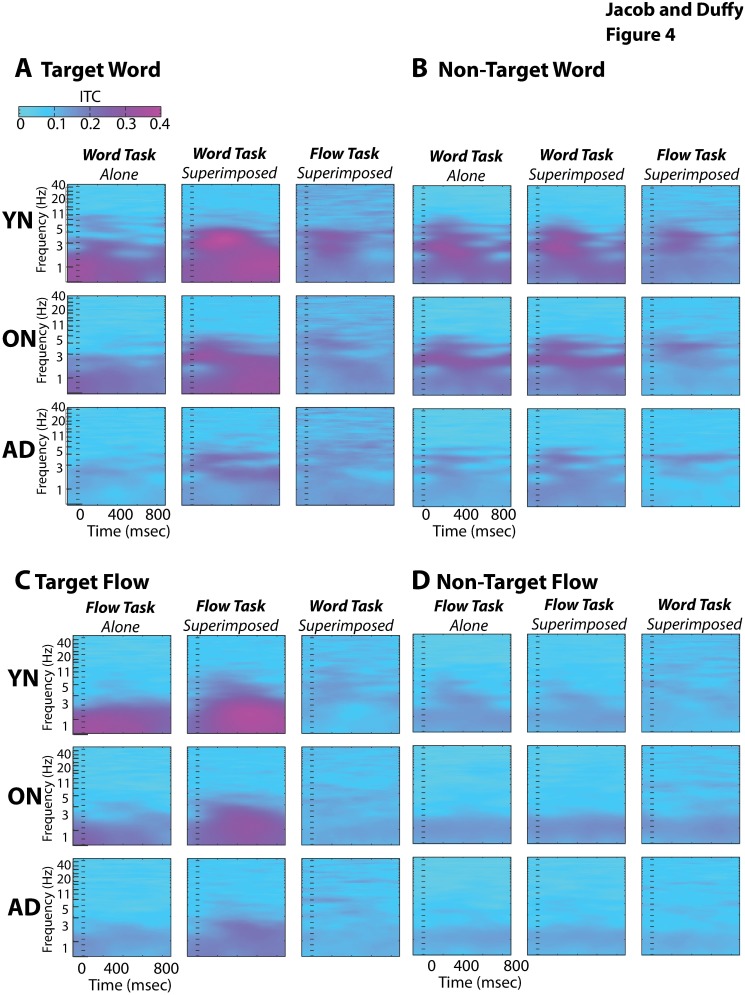
Inter-trial coherence (ITC) of recorded responses. Mean ITC of response phase (color) elicited across frequencies (ordinate) and the time-course of the 1.11 Hz stimulus cycle (abscissa). Phase coherence for target word (A) and flow (C) stimuli is most pronounced for superimposed stimuli during the word task (middle), relative to the word alone stimulus (left), or superimposed stimuli during the flow task (right). ITC for non-target word (B) and flow (D) stimuli show similar effects.

Inter-trial coherence (ITC) in the 0–2 Hz range decreases across subject groups (group F2, 421 = 24.72, p<.001, THSDs YN > ON > AD). ITC is also affected by condition and stimulus, the strongest ITCs elicited with task-irrelevant 2^nd^ stimuli, especially for flow (condition-by-stimulus F2, 421 = 88.49, p<.001). ITCs in the 2–7 Hz range of the spectral harmonics, only show group difference in YNs (F2, 421 = 21.24, p<.001, THSDs YN > ON = AD). These too are affected by condition and stimulus, especially with words in the word task (condition-by-stimulus F2, 421 = 88.49, p<001). These analyses show 2^nd^ stimulus and task effects, with the largest responses to superimposed stimuli, and the smallest responses to task-irrelevant stimuli, all most evident in YNs. (Figure 4A and B).

The ITCs evoked by non-target stimuli show the same stimulus, task, and group effects as the targets, but without effects of task-irrelevant 2^nd^ stimuli. These effects are most prominent in YNs for word stimuli in the 0–2 Hz range (stimulus F1, 416 = 17.46, p<.001, THSDs word > flow stimuli; condition F2, 416 = 5.60, p = .004, THSDs word > flow tasks; group F2, 416 = 7.89, p<.001, THSDs YN > AD; condition-by-stimulus F2, 416 = 16.73, p<.001, THSDs word alone and combined > all others; group-by-stimulus F2, 416 = 10.15, p<.001, THSDs YN word > all others) with the same pattern of significant effects obtained for the 2–7 Hz range (all p<.001, THSDs as for the 0–2 Hz). (Figure 4C and D).

In sum, task-irrelevant 2^nd^ stimuli greatly enhance target word and flow coherence with little effect on spectral power. In contrast, changing tasks greatly diminishes coherence, as well as decreasing power. Like task effects, aging and AD effect coherence and power, with successive decreases in coherence, but a paradoxical increase in power with aging, that is lost in AD.

### Relating Neurophysiologic and Behavioral Measures

We explored relations between neurophysiological measures and task performance, focusing on ADs as the only group with substantial variability in performance. Multiple linear regression identified variables predicting percent correct responses in the word and flow selective attention conditions.

Word task accuracy (R^2^ = 0.758, F3, 19 = 20.6, p<0.001) relates to phase coherence (β = 1.31), with small contributions from the word spectral fourth harmonic (β = 0.028) and P3 amplitudes (β = 0.002). Similarly, flow task accuracy relates (R^2^ = 0.605, F3, 19 = 11.4, p<0.001) to phase coherence (β = 1.12), with small contributions from the third harmonic (β = 0.023), and P3 amplitudes (β = 0.0012). Thus, we find that phase coherence is, by far, the best predictor of selective attentional task performance in AD.

## Discussion

### Mechanisms of Selective Attention

ERPs to task-related words and flow are enhanced by superimposing a task-irrelevant 2^nd^ stimulus, potentially related to increased phase coherence. Previously, task-irrelevant stimuli were seen to reduce responses to task-relevant stimuli. That distractor inhibition [45], [46], [47] is smaller with complex stimuli and larger with demanding tasks [48], [49], [50]. Our selective attention task reverses that effect, with the 2^nd^ stimulus enhancing responses (Figure 2).

The attentional control of visual processing has been linked to fronto-posterior signals seen as late ERP components (N2b, N2pc, etc.) [51], [52]. These late components are lost when distraction [45], [53], [54] blocks frontal stimulus selection [55], [56]. Our 2^nd^ stimuli do not distract our subjects, performance is not impaired, or block late ERPs. This may reflect the independence of ventral extrastriate word processing and dorsal extrastriate flow processing [57], [58], controlled by parallel, reciprocal, fronto-posterior pathways.

The neural mechanisms of selective attention may be revealed by phase coherence increases when adding task-irrelevant 2^nd^ stimuli, with increased coherence (Figure 4) potentially reflecting phase locking on the task-relevant stimulus stream [59]. Phase locking could create fronto-posterior resonance, promoting the frontal propagation of task-relevant visual input [60], [61], [62]. In naturalistic circumstances, phase locking could be linked to intrinsic posterior cortical rhythms [63], [64], [65], [66]. In our studies, phase locking is seen by synchronizing our analyses to the tagging frequency of the task-relevant stimulus.

### Distinguishing Aging and AD

Aging is thought to be associated with a degradation of top-down fronto-posterior control mechanisms [12], [13], [67], with effects that may be compounded by cortico-cortical disconnection in AD [68], [69], [70]. In our studies, such effects are seen as successive declines in attention dependent phase coherence in aging and AD (Figure 4). Our cross-sectional data do not support inferences about disease progression in individual subjects. However, across our subjects and groups, phase incoherence is the best predictor of attentional dysfunction, which is consistent with the prominent group differences in executive function (Table S1).

Paradoxically, aging causes an increase in total spectral power, whereas AD causes a still greater decrease (Figure 3). Mechanistically the differential effect of aging and AD on total power and phase coherence may be linked. That is, in aging, the loss of signal coherence might trigger an enhancement of the net neural activity evoked by that signal to boost the reliability of signal transmission. Such an increase in net activity of engaged neuronal populations would cause more neurons, to be more active, more of the time. That would be the case whether individual neurons in a circumscribed area are responding more, or whether more neurons are responding in or across networked areas, or both.

Our study does not support definitive inferences about the underlying pathophysiologies of aging, AD, or their potential inter-relations. However, our finding that cortical hyper-responsiveness is task-dependent, may favor mechanisms operating at the level of network dynamics. One scenario for compensatory task-dependent hyper-responsiveness might link signal incoherence to the recruitment of functionally overlapping neuronal populations to maintain task performance. This is consistent with our finding that the neurophysiological changes seen in aging are associated with success in the behavioral tasks, and with reports suggesting that older adults show greater neural activity than younger subjects when achieving comparable levels of task performance [12], [14], [71]. Neuro-behavioral compensation could be actively engaged by greater effort in older adults, or by a passive feedback control process. In fact, subjective effortfulness could reflect such a process; effort as neural recruitment [72].

Aging related cortical hyperactivity may contribute to the pathophysiology of AD [2], [3], [6], potentially triggering the transition from aging to AD [7]. These views are consistent with likely molecular pathophysiologies of AD, with increased total power generation in aging causing hyper-metabolic changes [73] that promote excito-toxicity [5]. Excito-toxicity could promote changes in the generation and processing of endogenous proteins that compose the plaques and tangles of AD [74]. This might further exacerbate network incoherence [75] and critically impair spectral power generation [76]. Thus, aging and AD are neurophysiologically distinguishable, and their association may be causal.

## Supporting Information

Table S1 **Demographic and neuropsychological profiles of subject groups.** Post hoc tests of group differences were performed using Tukey’s honestly significant differences (THSDs, P<0.05). For each measure, group differences that are not significant are included in the box frame; those that are significantly different are in different box frames.
(PDF)
