# Supplementary material for: Might Cortical Hyper-Responsiveness in Aging Contribute to Alzheimer’s Disease?
Source: PLoS One. 2014 Sep 10;9(9):e105962. doi: 10.1371/journal.pone.0105962 (PMC4160186; doi:10.1371/journal.pone.0105962)
Supplement: Table S1 — Demographic and neuropsychological profiles of subject groups. Post hoc tests of group differences were performed using Tukey’s honestly significant differences (THSDs, P<0.05). For each measure, group differences that are not significant are included in the box frame; those that are significantly different are in different box frames. (PDF) [file pone.0105962.s001.pdf]

Jacob and Duffy
Table S1

	YN	ON	AD	
Number of Subjects	21	20	14	
Sex	10 M, 11 F	13 m, 7 F	9 M, 5 F	1 way ANOVA
Mean Age	21.5 +/- 0.6	77.0 +/- 1.8	73.6 +/- 2.1	p < 0.001
MMSE	29.8 +/- 0.1	28.6 +/- 0.3	27.0 +/- 0.7	p < 0.001
Road Map	31.2 +/- 0.3	29.3 +/- 0.6	28.6 +/- 1.1	n. s.
Figural Memory	8.2 +/- 0.4	6.8 +/- 0.3	5.1 +/- 0.4	p < 0.001
Immediate Verbal	21.4 +/- 0.7	15.6 +/- 0.7	9.9 +/- 1.3	p < 0.001
Delayed Verbal	7.8 +/- 0.2	5.9 +/- 0.3	3.1 +/- 0.5	p < 0.001
Animal Naming	25.9 +/- 1.8	19.0 +/- 0.9	15.6 +/- 1.3	p < 0.001
FAS	48.8 +/- 3.5	38.1 +/- 2.2	37.5 +/- 2.0	p < 0.001
Line Orientation	25.6 +/- 1.4	26.2 +/- 0.9	22.7 +/- 1.8	n. s.
Facial Recognition	47.9 +/- 1.0	47.1 +/- 1.1	43.6 +/- 1.9	n. s.
Trails A	Not Tested	33.7 +/- 2.3	89.3 +/- 24.6	p < 0.01
Trails B	Not Tested	86.0 +/- 8.9	302.8 +/- 98.7	p < 0.01
